# Compliance With a Biopsychosocial Assessment Template When Assessing Presentations of Self-Harm or Suicidal Ideation by Liaison Psychiatry Practitioners in Dorset Healthcare: A Clinical Audit

**DOI:** 10.1192/bjo.2024.536

**Published:** 2024-08-01

**Authors:** Aryn Azlan, Kirstie Gale

**Affiliations:** University Hospitals Dorset, Poole, United Kingdom

## Abstract

**Aims:**

To evaluate whether a comprehensive biopsychosocial assessment is performed for patients presenting with self-harm or suicidal ideation in clinical practice, following National Institute for Health and Clinical Excellence guidelines 225 (NG225). We assessed Dorset Healthcare Liaison Psychiatry practitioners' compliance with a standardized biopsychosocial assessment template.

**Methods:**

A standardized biopsychosocial assessment template, aligned with NG225, is utilised in all Dorset Healthcare Liaison Psychiatry services for conducting initial assessments. Included data were the initial assessments of adult patients presenting from 01/08/2023 to 30/09/2023 for the following indications: 1) a suicide attempt, 2) a self-harm incident, or 3) suicidal ideation. Any initial assessment that did not use the standardised template was excluded. Retrospective analysis of Rio records assessed compliance with each heading on the biopsychosocial assessment template.

**Results:**

A total of 60 records were included from Dorset Healthcare Psychiatry Liaison Services. Only one heading, the “Presenting Situation”, was documented in all assessments (100%). Psychiatric headings on the template showed high compliance: “Mental State Examination” and “Risk Summary” were each documented in 98% of assessments, and “Psychiatric Formulation” in 92%. The “Carer/Parent's Understanding of the Assessment” was the least assessed (40%). Other significant headings that showed moderate compliance were, “Safeguarding Concerns” (71%), “Physical Health History” (75%) and “Social Situation” (81%).

**Conclusion:**

Our findings emphasize the need for more comprehensive biopsychosocial assessments in Dorset Healthcare Liaison Psychiatry services. While Liaison Psychiatry practitioners exhibit proficiency in evaluating psychiatric aspects, there is reduced compliance in assessing social aspects, notably in assessing family understanding. Future qualitative analyses will evaluate practical barriers and human factors affecting compliance with specific headings. Moreover, data collection can expand to encompass additional Mental Health services in a wider catchment area, including settings such as community and inpatient facilities.

